# A thematic analysis of shared experiences of essential health and support personnel in the COVID-19 pandemic

**DOI:** 10.1371/journal.pone.0282946

**Published:** 2023-03-20

**Authors:** Linda Carman Copel, Suzanne C. Smeltzer, Christine D. Byrne, Mu-Hsun Chen, Donna S. Havens, Peter Kaufmann, Heather Brom, Jennifer Dean Durning, Linda Maldonado, Patricia K. Bradley, Janell Mensinger, Jennifer Yost

**Affiliations:** 1 M. Louise Fitzpatrick College of Nursing, Villanova University, Villanova, Pennsylvania, United States of America; 2 Department of Mathematics and Statistics, Villanova University, Villanova, Pennsylvania, United States of America; 3 School of Nursing, Massachusetts General Hospital Institute of Health Professions, Charlestown, Massachusetts, United States of America; 4 Department of Clinical and School Psychology, Nova Southeastern University, Fort Lauderdale, Florida, United States of America; University of Auckland, NEW ZEALAND

## Abstract

**Aims and objectives:**

Studies have shown that the COVID-19 pandemic has taken a toll on individuals who interact with patients with SARS-CoV-2 but focused largely on clinicians in acute care settings. This qualitative descriptive study aimed to understand the experiences and well-being of essential workers across settings during the pandemic.

**Background:**

Multiple studies of the well-being of individuals who have cared for patients during the pandemic have included interviews of clinicians from acute care settings and revealed high levels of stress. However, other essential workers have not been included in most of those studies, yet they may also experience stress.

**Methods:**

Individuals who participated in an online study of anxiety, depression, traumatic distress, and insomnia, were invited to provide a free-text comment if they had anything to add. A total of 2,762 essential workers (e.g., nurses, physicians, chaplains, respiratory therapists, emergency medical technicians, housekeeping, and food service staff, etc.) participated in the study with 1,079 (39%) providing text responses. Thematic analysis was used to analyze those responses.

**Results:**

Four themes with eight sub-themes were: Facing hopelessness, yet looking for hope; Witnessing frequent death; Experiencing disillusionment and disruption within the healthcare system, and Escalating emotional and physical health problems.

**Conclusions:**

The study revealed major psychological and physical stress among essential workers. Understanding highly stressful experiences during the pandemic is essential to identify strategies that ameliorate stress and prevent its negative consequences. This study adds to the research on the psychological and physical impact of the pandemic on workers, including non-clinical support personnel often overlooked as experiencing major negative effects.

**Relevance to clinical practice:**

The magnitude of stress among all levels of essential workers suggests the need to develop strategies to prevent or alleviate stress across disciplines and all categories of workers.

## Background

The COVID-19 pandemic is an unprecedented and ongoing global public health crisis. As of October 4, 2022, a total of 96,249,466 COVID-19 cases have been reported in the United States [1]. The COVID-19 pandemic has affected all aspects of life, especially for frontline healthcare workers [2] who are exposed to overwhelming pressure with consequential psychological stress [3].

Qualitative researchers have shown that the COVID-19 pandemic has increased healthcare workers’ workload [4–8]. Additionally, frontline healthcare workers lacked essential resources and personal-protective equipment (PPE) required to safely perform their job duties and provide patient care [9–11]. Along with workload and resource-related challenges, the persistent physical and emotional demands of caring for patients during a pandemic have exacerbated previous barriers to workplace well-being [12]. Healthcare workers have experienced high levels of burnout and psychological distress [13–20] as well as adverse physical effects during the COVID-19 pandemic [21–23]. Globally, the numerous challenges caused by COVID-19 have led to increased mental health issues, such as anxiety and depression [12, 24, 25] among healthcare workers [26]. Healthcare workers have experienced post-traumatic stress disorder (PTSD) [27–29] and suicidal ideation [16, 30, 31].

The COVID-19 pandemic has affected the U.S. healthcare system and its workers significantly. A poll from September 2021 completed by 1,000 U.S. healthcare workers revealed that since February 2020, increasing numbers of healthcare workers have either quit (18%) or lost their jobs (12%) during the pandemic, while 19% have considered leaving their employer or the healthcare field entirely [32]. Additionally, some healthcare workers have been fired or resigned over vaccination mandates [33] or have developed “long-COVID” and can no longer work [34]. The American Association of Critical-Care Nurses (ACCN) surveyed more than 6,000 acute and critical-care nurses and found that 66% of nurses have considered leaving nursing after their pandemic experiences, while 92% believe the pandemic has exhausted nurses and will cut their careers short [35]. As healthcare workers are leaving the frontlines, critical staffing shortages are overwhelming hospitals in the United States, which may intensify workplace challenges encountered by frontline healthcare workers who report relentless emotional and physical exhaustion [34, 36, 37].

This study was conducted to obtain a comprehensive understanding of the experiences of essential workers, including healthcare workers, support personnel, and first responders, during the COVID-19 pandemic. This study provides a description of the experiences of essential workers working in healthcare facilities or in the community during the COVID-19 pandemic from May 2020 –June 2021 in the United States.

## Methods

This qualitative descriptive study is part of an effort to explore the effects of the COVID-19 pandemic on essential workers employed in settings and interacting with individuals infected with coronavirus (SARS-CoV-2). The study, the COVID-19 Study and Registry of Healthcare and Support Personnel (CHAMPS), was approved by the Villanova University Institutional Review Board (IRB) and conducted through the Internet. Written informed consent was obtained from each participant prior to enrolling in the study and was approved by the IRB. An invitation describing the study was sent by email to essential workers through websites of professional organizations, alumni societies, news media, professional and trade groups, social media, and healthcare facilities that treated patients with COVID-19. The CHAMPS study was initiated in the early acute phase of the U.S. pandemic in the spring of 2020 [38].

Data were collected for the CHAMPS study using an online survey that asked for demographic and professional characteristics, and included measures of anxiety, depression, social support, traumatic distress, and insomnia. Participants were invited to provide a free-text response to the following: *“If you wish*, *please describe your experiences during the COVID-19 pandemic in as many words as you need*.*”* Data analyzed for this paper are limited to the responses to the free-text open-ended survey item.

For the purposes of this study, "essential worker" was defined as those directly or indirectly working with individuals with confirmed or suspected COVID-19, for example, healthcare personnel, support personnel, and first responders [38]. Participants were adult workers ages 18 years or older who self-identified as essential healthcare personnel (e.g., physicians, nurses, phlebotomists, respiratory therapists, pharmacists), support personnel (e.g., laboratory staff, food service), or first responders (e.g., police officers, firefighters, emergency medical technicians, paramedics) working in any healthcare facility or in the community. Enrollment occurred over 13 months, from May 2020 through June 2021, in the United States.

A total of 2,762 essential workers participated in the CHAMPS study and 1,079 (39%) of them provided a response to the open-ended survey item. These responses from the participants were downloaded to a separate text file that allowed the research team to analyze the free-text responses.

Thematic analysis [39] was used to analyze the responses. The first three authors read all text responses to identify meaningful statements provided by participants. Next, the researchers organized and coded those statements with labels that reflected the responses. Throughout the coding process the researchers focused on identification of issues, similarities and differences revealed in the participants’ responses. The researchers met to reflect on the data during the analysis process. Coding was performed by hand on each text response, and each researcher’s individual codes were found to be representative of the participants’ responses.

Three members of the research team met to discuss the preliminary codes or labels and came to consensus on them. The researchers then went back to the responses and categorized meaningful statements they identified that were consistent with the codes. At subsequent weekly meetings over a period of several months, the team discussed examples of meaningful statements and together revised or refined codes. The themes were organized from the codes with a focus on the participants’ quotes. In the process of developing the themes, the researchers worked with the codes in a comprehensive and meaningful way while being faithful to the data. With further discussion, four themes and eight subthemes were identified. Trustworthiness and rigor of the study were addressed by the interaction and collaboration among the researchers throughout the process and in several research team meetings. Since responses were anonymous, member checking was not possible. The researchers followed the COREQ guidelines [40] in this study.

### Sample

The demographic characteristics of the 1,079 participants are described in Table 1. Most participants were female (85%, n = 917), White non-Hispanic (85.7%, n = 925), and engaged in clinical or support service activities involving direct care of patients with diagnosed or suspected COVID-19 (86.9%, n = 937). Almost 75% of participants worked in hospital settings during the pandemic (large/metropolitan hospitals (35%, n = 378), suburban/regional hospitals (27.4%, n = 296) and rural/community hospitals (10.8%, n = 116) and had various job roles. All the job categories self-identified by the participants are included in Table 1.

**Table 1:** Demographics.

Variables	N	(%)
**Age (Mean ±SD)**	42.65±13.32	
**Gender**		
Female	917	(85.0)
Male	158	(14.6)
Gender Non-Conforming/Other	4	(0.4)
**Race**		
White/Non-Hispanic	925	(85.7)
Black/African American	41	(3.8)
Latinx/Hispanic	37	(3.4)
Asian/Pacific Islander	34	(3.2)
Multi-racial/Mixed ethnicities	23	(2.1)
Other	15	(1.4)
Native American/Alaska Native	4	(0.4)
**Relationship**		
Married/Domestic partnership	660	(61.2)
Single	309	(28.6)
Divorced	75	(7.0)
Widowed	18	(1.7)
Separated	14	(1.3)
No response	3	(0.3)
**Living situation**		
I live with spouse/partner/significant other	373	(34.6)
I live with spouse/partner/significant other and dependent children	356	(33.0)
I live alone	141	(13.1)
I live with a roommate/multiple roommates	67	(6.2)
I live with parent(s)	62	(5.7)
I am a single parent living with dependent children	45	(4.2)
Other living arrangement not listed	25	(2.3)
I live with siblings	10	(0.9)
**Education**		
Bachelor’s degree	478	(44.3)
Master’s degree	295	(27.3)
Associates or Technical degree	121	(11.2)
Doctoral degree (e.g., JD, MD, PhD)	95	(8.8)
Some college or trade school	74	(6.9)
High school degree or equivalent (e.g., GED)	13	(1.2)
No response	2	(0.2)
Less than high school degree	1	(0.1)
**Region of Workplace**		
**N**ortheast	696	(64.5)
Midwest	207	(19.2)
West	72	(6.7)
Southwest	65	(6.0)
West	37	(3.4)
Puerto Rico	1	(0.1)
Unknown	1	(0.1)
**Please describe the kind of setting in which you are MOST FREQUENTLY working due to the pandemic.**	207	(19.2)
Large/metropolitan hospital	378	(35.0)
Suburban/regional hospital	296	(27.4)
Rural/community hospital	116	(10.8)
Ambulatory/outpatient care (i.e., seeing patients face-to-face)	86	(8.0)
Work takes place in the field (such as fire or police department roles)	73	(6.8)
Long-term care facility	67	(6.2)
Other setting not listed (e.g., telehealth)	39	(3.6)
Home healthcare	24	(2.2)
**Which category below BEST describes your job role as a COVID-19 essential worker?**		
Nurse (e.g., including RN, CRNP, CRNA, Nursing Assistant, LPN)	631	(58.5)
Other*	148	(13.7)
Emergency Medical Technician (including EMTs, AEMTs, and EMRs)	49	(4.5)
Physician	39	(3.6)
Paramedic	36	(3.3)
Social Work	25	(2.3)
Physical Therapist	22	(2.0)
Aide	19	(1.8)
Respiratory Therapist	19	(1.8)
Reception/Unit Clerk/Administrative Assistant	16	(1.4)
Occupational Therapist	12	(1.1)
Psychologist/Therapist/Licensed Professional Counselor	10	(0.9)
Pharmacist	9	(0.8)
Firefighter	8	(0.7)
Care Coordinator/Case Manager	7	(0.7)
Physician Assistant	7	(0.7)
Registered Dietician	7	(0.7)
Medical Assistant	4	(0.4)
Food Services	3	(0.3)
Pharmacy Technician	3	(0.3)
Facilities	2	(0.2)
Police Office	2	(0.2)
Phlebotomist	1	(0.1)
*Job Category Other (n = 148)		
Allied Health Professional	27	(18.2)
Chaplain	25	(16.9)
Manager/Coordinator	21	(14.2)
Administrator/Administration	19	(12.8)
Imaging Technician	15	(10.1)
Nursing Specialty Role	15	(10.1)
Health Care Technician	12	(8.1)
Community Service Worker	8	(5.4)
Speech and Language Therapist	7	(4.7)
**Are you directly engaged in clinical or support service activities involving the diagnoses and/or care of patients with, or suspected to have COVID-19?**		
Yes	937	(86.9)
No	142	(13.1)

## Results

Four themes and eight sub-themes were identified through thematic analysis of the responses to the open-ended survey item. The themes were: Facing hopelessness, yet looking for hope; Witnessing frequent death; Experiencing disillusionment and disruption within the healthcare system, and Escalating emotional and physical health problems. Participants’ statements were extracted for each theme and subtheme to capture their views, feelings, and personal situations.

### Theme 1 Facing hopelessness, yet looking for hope

The theme, facing hopelessness, yet looking for hope, represented the pervasive feeling that the overwhelming challenges of caring for patients with COVID-19 felt insurmountable at times. Yet the essential workers verbalized that despite “the physical and mental exhaustion from extra duties and responsibilities, there was hope for the future.”

### Subtheme 1 Feeling despair

A sense of despair was described by a number of respondents. A feeling of “being unprepared,” “ever-changing CDC and hospital guidelines,” and “lacking adequate protective gear,” resulted in frontline workers’ feelings of hopelessness. Often, they equated their “extreme sadness,” “moral distress,” and “being in a state of continued fearfulness,” with “living in a state of despair.” Additional responses reflecting despair include: “First responders like me have day after day of high anxiety and things going wrong, and it’s only getting worse out here” and “We do what we do every day but wonder if it will make a difference.” Additionally, essential workers employed by hospitals wrote about the difficulty of “going to work each day… and telling a family they can’t come to the hospital while knowing they will never see their loved ones again.” Other respondents stated, “I have seen so much suffering…” and “my patients fought and found so many reasons for staying alive, but eventually they died.” A nurse described the current experience: “It is beyond draining and working with patients with COVID makes you, as a nurse, feel helpless, exhausted, depressed and burnt out.”

### Subtheme 2 Searching for positives

While there were many statements by essential workers expressing how hopeless they felt, they were also hopeful, stating, “We looked for reasons to be hopeful” and “We worked hard with the hope that we were able to make a difference for patients in these difficult times.” Although respondents shared that they were “pulled in many directions,” “feeling irritable and numb,” and struggling with “compassion fatigue,” they expressed how colleagues in the field and staff on the units “rose to the occasion with grace, dignity and courage, and provided excellent care… even when we felt we were fighting a losing battle,” and “It felt like controlled chaos… because I work with a great team which made it bearable.” One respondent wrote: “As nurses, we understood each other and supported each other to make a difference with the patients we cared for.” Another stated, “I reminded myself and others that there is something good in every day, so look through a lens of gratitude that shifts negativity into optimism and energy to help others and hope for the future.” A paramedic reported, “COVID brought huge changes to my work and personal life,… and I learned to connect with others at different and stronger levels than before.” One respondent stated, “I am a nurse, and it is my duty to help the sick. I am proud and hope to be a part of the solution for treating patients with COVID-19.”

### Theme 2 Witnessing frequent death

This theme, witnessing frequent death, addressed the high number of deaths experienced by frontline workers along with the distress they experienced with patients being alone as they were dying. Being present for multiple deaths of patients with COVID-19 was described by many respondents who often witnessed disturbing deaths. These experiences were extremely distressing to many study respondents.

### Subtheme 1 Disturbing deaths

“Witnessing people die both inside and outside of clinical settings became a daily recurrence,” a chaplain reported. Nurses working in intensive care units (ICU), COVID units, emergency departments, medical-surgical units, long-term care, and other health facilities, conveyed that “more patients died from COVID in the first few weeks than I have ever seen in my entire nursing career.” A nurse who worked for over 30 years in SICU/Trauma described “numerous codes that still ended in patient death.” One nurse noted, “There were 18 deaths on the unit with 16 codes in one day.” Another acknowledged,

There were between 1 to 7 codes a day and even more deaths from COVID-19. Our doctors often tried to convince family members to make their loved one a DNR (Do Not Resuscitate) status, since the patients we coded, lingered three to four more weeks, and then died.

A nurse manager stated, “The number of patients who traumatically died one after another was surreal…and no one is meant to see this.” A nursing assistant commented, “People were dying all the time; we ran out of body bags.”

Patient deaths were described as “disturbing,” “awful,” and “never being able to erase from our memories the horrible deaths we experienced.” A nurse stated, “Caring for patients suffering, going unresponsive, and dying from severely labored breathing and intense air hunger was extremely traumatic.” Nurses participated in caring for patients “…on life support for way too long and on high vent settings with no chance at a meaningful recovery.” A nurse explained,

Family members were admitted and intubated side by side. Members of the same family passed away days apart even after doing every intervention imaginable, but with no medical intervention able to save them. Some patients’ temperatures skyrocketed to 107℉… there were times I witnessed four patients coding at the same time. Patients reached out their hand and I gave them mine in return to let them know they were not alone.

### Subtheme 2 Experiencing the trauma of patients dying alone

Various essential workers in the community and the hospital spoke about their painful experiences of watching people “undergo the ordeal of dying without loved ones present.” Nurses addressed that “it was terrifying to watch the fear in patients’ eyes as they were dying alone without family members.” An emergency department nurse explained, “We were working in warlike conditions… intubating multiple patients at one time, making elderly patients DNRs/DNIs (Do Not Intubate) on arrival because aggressive medical management was futile… and knowing how awful it was that they were dying alone.”

Nurses providing care on COVID-designated units related that, “Every day I watched my patients die alone with no family at the bedside;” “We, not their family members, were often stroking their hair or holding their hands as they died alone;” and “Watching patients die without family and suffer when there is nothing more we could do, will haunt me for the rest of my career.” Another nurse added, “Patients die alone. Families are Face Timing their goodbyes and not able to be there… These experiences I am having trouble dealing with and this will stick with me for a long time.” Two funeral directors commented that “it was devastating for families to have loved ones die alone,” and “I have nightmares about how the people in my care died alone.”

### Theme 3 Experiencing disillusionment and disruption within the healthcare system

The third theme, experiencing disillusionment and disruption within the healthcare system, reflected essential workers’ vulnerability and their increasing disillusionment with healthcare systems and government officials. Their disillusionment was increased by lack of sensitivity of administrators to the very disturbing situations encountered or experienced by frontline workers and their impact on them.

### Subtheme 1 Prevailing mismanagement and disorganization

Problematic mismanagement and disorganization in their healthcare settings and organizations were identified by respondents as very disturbing and adding to their stress. Issues that many nurses focused on were inadequate to non-existent communication about caring for patients with COVID, agencies not taking measures to protect staff and patients from the spread of the virus, and inadequate staff, PPE, and other resources. Working conditions described by many essential workers (including nurses, nursing assistants, paramedics, police officers) were characterized as “exhausting with no time for breaks/meals in order to get the job done.” One nurse expounded upon the situation:

Even after weeks of admitting patients with COVID the hospital was not prepared to handle these patients… because of the lack of PPE and fear of COVID, the only person who went into the room was the nurse and possibly a respiratory therapist. The doctor only went into the room once a day. The housekeepers, dietary, OT/PT/ST, social workers, etc., were not allowed into the rooms. The nurse was in the room constantly doing the work for all these specialties… The rooms were so small that the beds could barely fit and there was little to no room to get the vent, IV poles, or more than a couple of people into the room at one time. During a code, there was only myself and the respiratory therapist in the room, while the doctor was outside giving orders and other nurses were drawing up medications outside the room and handing them to me through the doorway.

The nurses were distressed by “the lack of information from administrators to staff.” Even when nursing staff asked about evidence-based practices, “we were not given answers to our questions and concerns.” One respondent explained:

Management was nowhere in sight during this time, and we felt incredibly unsupported. With so many people being out, we were extremely short-staffed and were working harder than ever. This did not seem to be noticed or appreciated. We were essentially on our own. We were all frightened and overwhelmed, but we just went on… Management never checked in on how we were doing emotionally. We watched coworkers spend weeks in the hospital, and some even die. There was minimal care for our overall mental well-being during and after, even still after all these months. Many nurses and ancillary staff have left, so we are still working short-staffed now.

Other staff focused on how appalled they were by the need to reuse PPE. “We are still reusing, and re-sterilizing used N95s, but we think just be grateful to at least have the N95s.” Several nurses shared concerns that management was “hiding supplies” and “rationing resources.” A nurse described the situation by stating:

The moment-to-moment changes coming down from management felt disorganized and added to the stress of taking care of our patients. The divide between beside staff and management has only deepened because of their disorganization and overall poor planning. It has also made working relations between my team brittle and tense. I felt let down by the healthcare system.

Although most respondents identified mismanagement, it is important to note that a few respondents reported that their hospital administrators and nursing managers did a credible job and indicated that they were prepared and had the necessary resources. One stated, “Unlike what I’ve heard about other hospitals, ours has been preparing for a pandemic since the Ebola outbreak. I am grateful for my administration’s foresight.”

### Subtheme 2 Lacking sensitivity and concern for the caregivers

A lack of sensitivity and concern for essential workers on the part of administration was described by respondents. A paramedic, for example, verbalized being “overlooked,” a dietician reported being “unsupported,” and a nursing assistant felt “forgotten.” Comments from a nurse coordinator, ER technician, and a respiratory therapist reflected the view that “hospitals are big business,” “the bottom line is hospitals want to make a profit… and staff safety is an afterthought.” One respondent stated, “Upper management does its best to pay lip service to our needs… we feel very disposable as nurses.” Another nurse added, “I was so concerned when I found out that my ED is providing us with ‘not for medical use’ masks and saying they are safe.” A physician assistant stated, “It bothered me that my hospital didn’t care about those of us working on the frontlines.” A nurse manager stated, “Decisions were based on what was best for the healthcare system when reassigning staff without a thought to the staff/patient exposure. Management stated, ‘I just need a body and you are that person.’” Another nurse asserted, “The hardest part for me is I do not feel my voice as a nurse is being heard, or my needs are being met by nurse managers or corporate leaders.” A respiratory therapist stated succinctly, “Our employer does not have our backs.”

### Theme 4 Escalating emotional and physical health problems

The last theme, escalating emotional and physical problems, addresses the physical and emotional exhaustion, exacerbation of health issues, fears and anxiety about these issues and their impact on frontline workers. Negative responses and behaviors of community members and society as a whole further increased frontline workers’ emotional and physical distress.

### Subtheme 1 Handling emotional and physical distress

Essential workers described themselves as “physically and mentally worn out.” They shared that they experienced “exhaustion that I never had before,” and indicated that “the more anxious and depressed I am, the more sleep problems I have.” Nurses, chaplains, and respiratory therapists disclosed that health problems normally under control such as asthma, diabetes, hypertension, chronic fatigue syndrome, autoimmune disorders, and dermatological conditions were exacerbated. They spoke of having “daily bouts of anxiety,” “panic attacks,” “stress headaches,” “shortness of breath during nightmares,” “terrifying fears of getting COVID and transmitting it to family members,” “crying daily after going home from work,” and “post-traumatic stress disorder.” One nurse described the distress experienced as being at a very high level in that “I realize that I was overwhelmed to the point of sometimes having suicidal thoughts and recognizing I had to see someone.” Respondents who were diagnosed with COVID-19 spoke of its “lingering effects” and “experiencing post-virus syndrome.”

Numerous respondents addressed being newly diagnosed with hypertension, anxiety, panic attacks, depression, and post-traumatic stress disorder. Many described being prescribed anti-anxiety medication, obtaining support through Employee Assistance Programs (EAP), and attending psychotherapy sessions. Several respondents indicated that it helped to talk about the horror, pain, and moral distress they experienced. One stated, “Talking to someone made me feel like I was living less and less in a nightmare.” Another stated that therapy “helped me work through my feelings of helplessness, and address feeling overwhelmed, and being pulled in too many directions.”

A nurse stated that she “pushed my emotions aside to focus on doing my job to the best of my ability.” A physician noted, “The need to hold back my feelings at work added to my stress level and made me more fragile and reactive to small slights in my home life.” Another respondent shared, “Every time I hear the words ‘patient with COVID-19,’ I become tachycardic and my heart sinks to my stomach, but I had to learn to say, ‘I’m ready for this, I’ve got this.’” Many essential workers gave credit to “their amazing healthcare workers who have transcended their roles, acting as family to all those in need and as a lifeline for all of us.” A nurse elucidated, “Coworkers have come closer together… and helped each other cope during this pandemic… because we truly feel that we are the only people who understand each other and what we are going through.”

### Subtheme 2 Feeling society’s disengagement

The term healthcare heroes did not last very long according to many respondents. One stated, “The public’s response to the virus has been extremely disheartening. We’re heroes one minute, and the next minute people don’t even want to wear masks because they appeared to tire of the concept.” A nurse articulated, “It makes me angry that those not exposed to how bad COVID is, tend to think that this is fake and it’s not as bad as the news portrays it to be.” “Society says nurses are heroes in one sentence, and in the same sentence the attitude changes.” “We are disrespected because people don’t follow the rules, don’t wear masks… and sound off about their personal freedom to do what they want to do and live the way they want to live.”

One nurse declared, “I feel disrespected by the federal government and have lost faith in the CDC.” Another respondent added, “There is a lack of compassion for others on a daily basis… it feels like chaos at work and in the world.” “It is disheartening to know that everyone is not on the same page.” Several other respondents criticized how poorly the pandemic was being handled by the federal government. One person stated, “I become angry watching politicians wasting time, money, and resources on other matters like impeachment… when people are suffering with COVID-19, unemployment, and the lack of a vaccine.” Another shared this comment, “The politicization of this pandemic caused a great rift in our response to it and hindered our ability to keep it managed to the best of our ability.” A nurse summarized the situation by stating,

I feel like we are fighting two battles, first, the disease itself, and second, the politics. People still do not believe it is real, our national leaders aren’t doing a damn thing about it. They worry about their own positions. The hospital leadership is out of touch with what is going on at the frontlines.

## Discussion

This study explored the experiences of essential workers from a variety of disciplines, working in multiple healthcare and community settings in the United States. Participants shared their written narratives in May 2020 –June 2021 as they were living and working during the COVID-19 pandemic. It was noted that the length of typed responses ranged from one or two words to 1,407 words. Most of the open-ended responses ranged from 274 to 536 words in length. (Typically, one typed page is appropriately 500 words single spaced.) This information is noteworthy, since essential workers who were engaged in caring for people with COVID-19, experiencing excessive workplace demands, and numerous emotions, took the time to write about their situations and express their feelings.

They felt very overwhelmed and reported hopelessness almost to the point of despair as they faced unprecedented and relentless challenges and expressed concern about the uncertainty of the future. Similar responses have been reported in other studies [4, 10, 19]. The stressful and chaotic situations frequently endured were out of the workers’ control. As participants were working tirelessly in their respective settings, they questioned if they were making a meaningful difference in their efforts, which also contributed to their sense of hopelessness.

Despite the hopelessness and despair the participants experienced; they expressed hope for more effective ways to care for individuals with COVID-19. Participants persevered, searched for hope, and identified some positive aspects of living and working though the pandemic. A sense of camaraderie and teamwork prevailed among workers, which has also been reported in other studies [8, 14, 41]. Also similar to other studies’ findings [18, 41–43] workers in our study identified self-care modalities, such as exercising, that assisted with coping.

Because of the toll of high stress levels on their health, new health problems among study participants developed and existing physical and psychological health issues intensified. Examples of physical conditions, such as asthma, diabetes, hypertension, chronic fatigue syndrome, autoimmune disorders, and dermatological conditions, were exacerbated. As their stress levels increased and were prolonged, some experienced daily episodes of anxiety leading to panic attacks, stress headaches culminating in migraines, bouts of shortness of breath, and terrifying nightmares. These findings reflect the interrelationship of physical symptoms and psychological distress [21] and are similar to those of other studies in which the study participants experienced physical symptoms and mental health issues [2, 10, 13, 22, 23, 27–29].

Experiences related to the deaths and dying of multiple patients with COVID-19 were particularly traumatic and disturbing for participants. This was especially evident among nurses working in hospital settings, as they watched patients in their care die alone without the presence or family or loved ones at the bedside [7, 44]. Other studies have identified the absence of family members at the bedside of acutely ill patients as distressing to healthcare workers even if the patient was not dying [45, 46]. The number of patients who died and the disturbing nature and circumstances of their deaths, without family members present, were particularly distressing to participants. Many felt ill prepared to provide end-of-life care and had little support, except from their co-workers, to cope with the situation. Researchers [47] described the moral distress of healthcare professionals concerned about end-of-life care, who were unable to allow family members at the bedside of dying patients because of imposed visitor restrictions.

Many participants in this study conveyed strong disillusionment with the administrators, the health care system, and the national political response to the pandemic. Although participants were dealing with inadequate PPE, inadequate staffing, lack of support for staff, and fear of contracting or spreading the virus, administrators were largely invisible, unavailable, unresponsive, noncommunicative, and seen as concerned more about the financial risks of healthcare systems rather than the well-being of the workforce. Participants also expressed disillusionment with many in their communities who questioned the seriousness of COVID-19 infection and ignored guidelines designed to minimize risks, such as use of masks and social distancing. Participants reported being ostracized by their communities because they worked with and provided care for patients with COVID-19, which has been reported in other studies [8, 48]. These responses further increased the stress of participants in the study.

An important point related to our data collection strategy is the unique method used to collect responses of essential workers during the COVID-19 pandemic. Data were collected through a single, open-ended survey item, that allowed participants to write about their own personal experiences. The open-ended survey item invited participants to write about any aspect of their experience during the pandemic that they wanted to share, which may have been similar to journaling for some participants as other researchers have found that expressive writing can help mitigate psychological distress [49]. During a time of uncertainty and stress, participants had the opportunity to write about their pandemic experiences and perceptions, as they were happening in real time. Other researchers have reported journaling to be a valuable self-care strategy during the COVID-19 pandemic [43] and an effective strategy to support home care workers [5].

It is also important to note that this method of data collection required participants to write their response, rather than talking about their experiences with another person (i.e., interview), which may have influenced the content and wording included in the responses. Similarly, other researchers [50] launched an anonymous website for community workers that provided an outlet for unprompted and uncensored stories of healthcare workers.

Several limitations of the study included the inability of the researchers to determine the experiences of participants in the CHAMPS study who did not respond to the open-ended survey item and the reasons they elected not to do so. It is unknown if the non-respondents had the same experiences or were too distressed to respond to the item, did not take or have the time to respond, or were fearful that expressing their personal situations may somehow become known to their employers. Since the survey was anonymous, it was not possible to conduct member checks. The study sample was primarily comprised of white females and lacked gender, ethnic, and racial diversity.

The notable strengths of the study included the large number of people (n = 1,079) who responded to the open-ended question and took the time to share their situations candidly, with extensive uninhibited disclosure of detail and without notable reservation. Participants had the freedom and the opportunity to express and convey their thoughts and feelings as they were living through the pandemic. The intensity and timeliness of the participants’ experiences were captured in their written narratives. This may be particularly true in those participants who wrote multiple pages of comments, and our method of data collection may have given voice to those who may not otherwise be heard.

## Conclusion

This study revealed major psychological and physical stress among essential workers employed in healthcare and community settings during the early phases of the COVID-19 pandemic in the United States, leading to despair and a sense of hopelessness. High levels of stress and distress resulted from uncertain and often overwhelming working conditions, lack of protocols for caring for patients with COVID-19, threats to participants’ physical safety and mental health, witnessing multiple disturbing deaths, absence of family members at dying patients’ bedside, lack of adequate physical and emotional support from administrators, and a sense of being abandoned by community members. The severity and prevalence of major psychological and physical stress experienced by essential workers during the COVID-19 pandemic may take years before healing and reconciliation can occur. Administrators’ recognition and appreciation of the highly stressful situations experienced by workers are essential to begin to repair the relationships that have been damaged or destroyed as a result of the negative experiences reported by participants in this study. Understanding highly stressful experiences during the pandemic is essential to identify and implement strategies that ameliorate stress and prevent its negative consequences, and to restore confidence and trust of administrators and the healthcare system on the part of healthcare personnel, support personnel, and first responders. Those affected need to be part of the process of developing interventions to address these issues now and in future pandemics. This study adds to the research on the psychological and physical impact of the pandemic on workers, including non-clinical support personnel often overlooked as experiencing major negative effects.

## Relevance to clinical practice

Naming and understanding the multitude and magnitude of highly stressful experiences during the pandemic are essential to building strategies that ameliorate stress and prevent its negative consequences. The experiences, exhaustion, and vulnerability of the participants who relentlessly continued to care for very ill patients diagnosed with the COVID-19 virus were not addressed by healthcare administrators. Awareness of the events and challenges that were occurring with essential workers during the pandemic need to be the focus of administrators in real time. The question, who cares for the caregivers? must be addressed and resources must be immediately available during times of crisis. The value of the healthcare and related workforce must be acknowledged through the actions of agencies and institutions to provide strong, effective, and equally important, visible leadership. The most salient characteristics of leadership desired by participants in this study during the COVID-19 pandemic were communication, collaboration, teamwork, flexibility, empathic understanding, and transparency. There was a strong need for involvement of all stakeholders as the healthcare environment experienced both clinical and operational transformation.

Preparation for potential health crises and possible future pandemics, as well as ensuring the availability of both internal and external resources based on the experiences described in this study is essential. Administrators must be actively involved in not only allocating resources for patient care and the safety of the workers, but also for ensuring the availability of relevant human resources required for the psychological and physiological well-being of all staff. Being present and continuing tangible demonstration of their support and commitment is meaningful to workers. This study adds to the identified psychological and physical impacts of the pandemic on essential workers, including non-clinical support personnel who are often overlooked, as experiencing profound negative effects from the on-going COVID-19 pandemic.
